# Comparison of Higher-Order Aberrations After Single-Step Transepithelial and Conventional Alcohol-Assisted Photorefractive Keratectomy

**DOI:** 10.4274/tjo.galenos.2019.14554

**Published:** 2020-06-27

**Authors:** Kemal Özülken, Çağrı İlhan

**Affiliations:** 1 TOBB ETU Medical School, Department of Ophthalmology, Ankara, Turkey; 2 Hatay State Hospital, Clinic of Ophthalmology, Hatay, Turkey

**Keywords:** Transepithelial photorefractive keratectomy, alcohol-assisted photorefractive keratectomy, asphericity, higher-order aberration, PRK

## Abstract

**Objectives:**

To compare the asphericity and higher-order aberration (HOA) outcomes of single-step transepithelial photorefractive keratectomy (tPRK) and conventional alcohol-assisted PRK (aaPRK) in patients with myopia and myopic astigmatism.

**Materials and Methods:**

Of the 108 eyes of 54 patients enrolled in the study, tPRK was performed on 54 (50%) eyes and aaPRK was performed on 54 (50%) eyes. The following parameters were compared: corrected distance visual acuity (CDVA), spherical equivalent (SE), flat and steep keratometry, intraocular pressure, central corneal thickness, asphericity, and HOAs including horizontal and vertical coma, horizontal and vertical trefoil, spherical aberration, second-order vertical coma, and aberration coefficient.

**Results:**

The demographic and baseline characteristics were similar between the two groups (p>0.05, for all). The aberration coefficient value was significantly lower in patients treated with aaPRK compared to patients treated with tPRK at postoperative 3 months, 6 months, and 1 year (p=0.022, p=0.019, and p=0.017, respectively). Differences in the other variables were statistically insignificant (p>0.05 for all).

**Conclusion:**

Both tPRK and aaPRK procedures obtain similar postoperative CDVA, SE, asphericity, and HOA outcomes, except the aberration coefficient value.

## Introduction

Transepithelial photorefractive keratectomy (tPRK) was described in the late 1990s as a two-step procedure to create a well-arranged epithelial wound edge. The corneal epithelium is removed with laser phototherapeutic ablation and then laser photorefractive ablation provides the desired refraction corrections.^1^ Unlike alcohol-assisted photorefractive keratectomy (aaPRK), using manual mechanical scraping or an alcohol solution is not needed in tPRK and there is no contact of any surgical equipment with the cornea. The lack of contact with the eye during the procedure is appealing to patients, who know this procedure as “no-touch laser” in Turkey.

Theoretically, the risk of epithelial defect and irregularity is minimal, but the predictability of this two-step unstandardized laser surgical procedure is limited due to a lack of adjusted nomograms. With old generation laser technology, this two-stage method was not used worldwide in the early period due to prolonged time to switch from phototherapeutic keratectomy (PTK) mode to PRK mode, corneal dehydration, and increased postoperative pain.^2,3^ Following developments in laser technology and improvements in algorithms over the years, a new modern variant of tPRK was described as no-touch laser tPRK in the Schwind Amaris platform (SCHWIND eye-tech-solutions GmbH, Kleinostheim, Germany).^4^ The most important methodological innovation in tPRK is the combination of PTK and PRK excimer laser applications in a single-step ablation procedure to remove the epithelium and stroma. This aspheric ablation profile is determined from literature data estimating the corneal epithelial thickness is 55 mm centrally and 65 mm peripherally. Recent studies show that the new generation single-step tPRK method reduces operative time, minimizes the epithelial defect area, eliminates the risk of toxicity on limbal cells because of the absence of alcohol, and causes less postoperative pain and corneal haze with faster healing time and visual recovery.^4,5,6,7^

Although there are many publications comparing the conventional aaPRK and single-step tPRK procedures, the tPRK method has undergone many minor modifications and nomogram adjustments over time. The aim of this study was to evaluate whether the latest version of the tPRK device is superior to that of aaPRK in patients with myopia and myopic astigmatism. The postoperative 1-year asphericity (Q value) and higher-order aberration (HOA) outcomes of tPRK and aaPRK were compared.

## Materials and Methods

### Design

This retrospective, nonrandomized, comparative study was conducted between January 2016 and June 2018 in the refractive surgery department of a private eye clinic, with approval granted by the local research ethics committee. All procedures were performed in accordance with the ethical standards of the Declaration of Helsinki for human subjects and written informed consent was obtained before surgery from each patient after a detailed explanation of the surgical procedures.

### Subjects

The study included patients aged over 18 years old with myopic or compound myopic astigmatism within the range of -1.00 to -8.50 diopter (D) manifest refraction spherical equivalent (SE), with better than 0.00 logMAR corrected distant visual acuity (CDVA) and stable refractive error for at least 12 months. Exclusion criteria were a history of ocular surgery, ocular trauma, or ocular disease, irregular astigmatism on corneal topography, estimated central stromal bed thickness less than 350 mm at the thinnest point, history of keloid formation, systemic disease that could affect corneal wound healing, and pregnancy. In total, 108 eyes of 54 consecutive patients were included in the study. Twenty-seven patients underwent tPRK and 27 underwent aaPRK according to patient preference. All subjects underwent bilateral refractive surgery performed by the same experienced and certified refractive surgeon (K.O.).

### Clinical Evaluations

Preoperative ocular and medical history was obtained and all preoperative examinations were performed after discontinuing soft contact lens use for at least 4 days. A detailed ophthalmic examination was performed by the same ophthalmologist. Manifest and objective refraction were determined and uncorrected distant visual acuity (UDVA) and CDVA were measured using Snellen chart. Decimal values were converted to logMAR for statistical analysis. Corneal tomography was performed with WaveLight^®^Oculyzer II (Pentacam, Oculus Optikgeräte GmbH, Wetzlar, Germany) and curvature, elevation, and thickness maps were obtained. Asphericity (Q value) calculations were obtained from the placido-based Allegrato Topolyzer version 1.59 (Alcon, Fort Worth, TX). Total corneal HOAs were analyzed, including horizontal and vertical coma (Z[3, 1] and Z[3, -1]), horizontal and vertical trefoil (Z[3, 3] and Z[3, -3]), primary spherical aberration (Z[4, 0]), second-order vertical coma (Z[5, -1]), and aberration coefficient in the Zernike analysis. The aberration coefficient is calculated from the value of the Zernike polynomial coefficients used to reconstruct the anterior corneal surface. If there are no abnormal corneal aberrations, aberration coefficient is 0.0; otherwise it becomes 1.0 or greater, depending on the degree of aberration.^8^ HOAs were evaluated in the 6.0 mm diameter central area with respect to the pupil center in a dark environment, and the pupil was not dilated.

Manifest refraction, UDVA and CDVA, intraocular pressure (IOP) measurement, anterior and posterior segment examination were done at postoperative 1 day, 1 week, 1 month, 3 months, 6 months, and 1 year. Corneal tomography evaluation, asphericity, and HOAs calculations were repeated at postoperative 3 months, 6 months, and 1 year. Postoperative 1 year was defined as the primary end-point of the study.

### Surgical Technique

A single experienced surgeon performed all surgeries using the same 6^th^ generation Amaris excimer laser version 750 S (Schwind Amaris, SCHWIND eye-tech-solutions GmbH, Kleinostheim, Germany). It was aimed to achieve emmetropia in all eyes.

In the operating room, topical proparacaine hydrochloride 0.5% (Alcaine, Alcon, Fort Worth, TX) was instilled for topical anesthesia and the eyelids were opened using a wire lid speculum. In the tPRK group, the epithelium was removed with excimer laser and the aberration-free tPRK ablation algorithm (SCHWIND eye-tech-solutions) was used. In the aaPRK group, the superficial epithelium was cut using an 8.5 mm diameter trephine and mechanically debrided with a spatula after exposure of the corneal surface to 20% ethyl alcohol solution for 10 seconds. Wavefront optimized ablation was performed according to the aberration-free algorithms calculated with the ORK-CAM software (version 4.63, SCHWIND eye-tech-solutions GmbH, Kleinostheim, Germany). Mitomycin C 0.02% was applied for 30 seconds in eyes with SE greater than 3 D and for 60 seconds if greater than 6 D due to increased risk of corneal haze.^9^ After laser ablation, a bandage contact lens (Senofilcon A [Acuvue Oasys, J&J, Vision Care, Inc., Jacksonville, FL]) was applied for 5 days. Postoperative topical moxifloxacin 0.5% (Vigamox, Alcon, Fort Worth, TX) 3 times a day for 1 week, topical dexamethasone (Maxidex, Alcon, Fort Worth, TX) starting after epithelial healing and tapered off over 3 weeks and artificial tears every 2 hours for 2 months were prescribed. No intraoperative or postoperative complications developed in any patient.

### Statistical Analysis

The data obtained from the study were analyzed using the Statistical Package for the Social Sciences (SPSS) 22.0 software (IBM Corp., New York, NY). Descriptive statistics were presented as mean ± standard deviations (SD) and minimum-maximum values. The normal distribution of the variables was tested using the Kolmogorov-Smirnov test. The non-parametric tests were used in analysis as the numerical data did not conform to normal distribution. The preoperative and postoperative variables of the same eye were compared using the Wilcoxon test. Postoperative asphericity and HOAs of the two groups were compared using the Mann-Whitney U test. Statistical significance was set at p<0.05 for all tests. Power and Sample Size (PASS) version 19 software (NCSS Statistical Software, Il, USA) was used for sample size and power calculations. It was found that at least 24 eyes were needed in each group for power of 80% (δ=6, σ=13, and alpha=0.05).

## Results

The tPRK group included 54 eyes of 27 patients (13 male, 14 female) with a mean age of 27.2±6.7 years (18-45 years). The aaPRK group included 54 eyes of 27 patients (12 male, 15 female) with a mean age of 26.1±6.2 years (18-43 years). There was no statistically significant difference in gender or age characteristics between two groups (p>0.05 for both).

**Figure 1:**
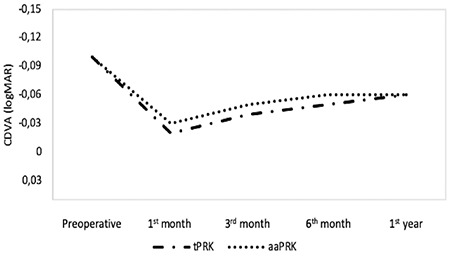
The mean corrected distant visual acuity (CDVA) in transepithelial photorefractive keratectomy (tPRK) and alcohol-assisted PRK (aaPRK) groups

**Figure 2:**
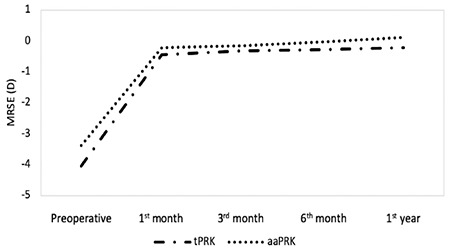
The mean manifest refraction spherical equivalents (MRSE) in transepithelial photorefractive keratectomy (tPRK) and alcohol-assisted PRK (aaPRK) groups

Preoperative clinical findings including CDVA, SE, flat and steep keratometry, IOP, and CCT values were similar in the two groups (p>0.05 for all). When comparing the postoperative 1-year measurements of the two groups, no significant difference was determined in CDVA, SE, flat and steep keratometry, IOP, and CCT (p>0.05 for all). No intraoperative or postoperative complications including haze, infection, undercorrections, overcorrections, or dry eye developed in any case. The preoperative and postoperative 1-year mean values of CDVA, SE, flat and steep keratometry, IOP, and CCT of the tPRK and aaPRK groups are presented in Table 1. Additionally, the mean CDVA and SE values are shown in Figures 1 and 2.

Asphericity and all HOAs were similar in the two groups at postoperative 3 months, 6 months, and 1 year (p>0.05 for all). The aberration coefficient differed significantly between the tPRK and aaPRK groups at postoperative 3 months, 6 months, and 1 year (p=0.022, p=0.019, and p=0.017, respectively). The postoperative results of asphericity and HOAs of the tPRK and the aaPRK groups are shown in Table 2.

## Discussion

Having excellent visual quality without using spectacles or contact lenses is the main rationale of refractive surgery. According to the results of this study, the postoperative CDVA and SE outcomes were highly satisfactory in both methods and there was no statistically significant difference between tPRK and aaPRK except in postoperative aberration coefficient value. However, visual quality is a very versatile concept and asphericity and HOAs are important factors affecting retinal image quality in patients who have undergone refractive surgery.^10,11^ It has been previously shown that excimer laser ablation increases ocular aberration in myopic eyes.^10,12^ The purpose of the present study was to compare the surgical outcomes of tPRK to aaPRK and to evaluate whether tPRK was superior to conventional aaPRK.

The profile used during tPRK is calculated based on data in the literature. As a result, the epithelial thickness of the central cornea is taken as 55 mm and the thickness of the epithelium in the 4 mm periphery as 65 mm. Moreover, the photoablative rate is set 20% higher than stroma.^13^ In the tPRK method, keratocyte apoptosis is restricted and a smooth uniform corneal surface is created with ideal epithelial regeneration.^14^ In contrast, an irregular ablation field and imperfect wound healing after mechanical epithelial removal with or without ethyl alcohol solution exposure can cause postoperative clinical or subclinical epithelial pathologies.^14,15^ In this regard, aaPRK and mechanical epithelial removal in PRK without alcohol give comparable outcomes.^16^

Since the tPRK method uses a standard epithelial ablation algorithm regardless of actual epithelial layer topometry, in some eyes less epithelial ablation than required is applied and an amount of ablation to be applied to the stroma may be applied to the remaining epithelium.^4^ Many studies have shown that there are differences between CCT and 3D epithelial maps.^5,6^ Therefore, refractive results and visual quality may be deteriorated after tPRK using a standard epithelial algorithm. In the current study, the groups were compared in terms of HOAs and only the difference in aberration coefficient value was found to be statistically significant in favor of aaPRK. It may be thought that the aberration coefficient is a general indicator that is affected by all HOAs and despite there being no difference in the individual HOAs, the aberration coefficient differed between the groups.^8^ Since aberration coefficient value was lower in the aaPRK patient group, we can conclude that corneas were more uniform after aaPRK and thus abnormal corneal aberrations were less common in this group. Moreover, in the light of this result, it can be said that the quality of vision after aaPRK is slightly better than after the tPRK method because the aberration coefficient is affected by all HOAs.

We did not find a statistically significant difference between the two groups when we evaluated HOAs. Kaluzny et al.^17^ evaluated the refractive results, predictability, safety, and efficacy of these two procedures and found that tPRK and aaPRK provided very similar results after a 3-month follow-up. Fattah et al.^18^ and Antonios et al.^19^ stated that the postoperative HOAs of two groups obtained by a Scheimpflug analyzer were similar. Luger et al.^5^ compared postoperative asphericity and HOAs of two groups using Pentacam HR and wavefront aberrometry and found no statistically significant difference after a 1-year follow-up period. To the best of our knowledge, ours is the first report to compare HOAs after tPRK and aaPRK using a Scheimpflug camera-based system and demonstrate different aberration coefficient results. The current study has filled this gap in the literature by having a long postoperative follow-up time and giving detailed measurements obtained at postoperative 1 months, 3 months, 6 months, and 1 year.

A disadvantage of tPRK is that the total excimer laser energy applied for epithelial removal and stromal ablation is higher than in the aaPRK method. The excimer laser energy can increase the temperature in the stromal tissue and cause postoperative haze formation.^20^ On the contrary, there are studies indicating that in aaPRK method more keratocyte apoptosis occurs because of the formation of bigger size of corneal epithelial defect and it provides more limbal cell damage due to use of alcohol and for these reasons aaPRK causes more corneal haze formation than the tPRK method.^20,21^ When all surgeries including tPRK and aaPRK in this study were considered, no difference was observed between the two groups in terms of postoperative haze severity, which can be attributed to the use of mitomycin C during the operation in both groups.

In the current study, there were no differences between the groups in terms of mean age or preoperative SE. Nevertheless, these parameters showed a wide range with large differences between maximum and minimum values (18 to 45 years and -1.00 to -8.50 D, respectively). This variability can directly affect postoperative outcomes. For example, the risk of corneal haze increases 2 fold in subjects with more than 6 D myopia.^9^ This should be considered an important limitation, and more homogeneous groups of patients in a narrower age range and separated into groups of low, moderate, or high myopia would be able to refine the results and overcome this limitation. Epithelial healing (re-epithelialization) processes and subjective visual parameters (pain, photophobia, photic phenomena, etc.) were not evaluated. In addition, there were no data about the mean operative times of the procedures in the current study; however, we observed that the tPRK method was shorter than the aaPRK method. Similar to these findings, there are studies indicating that the tPRK method performed with new generations of laser systems shortens the duration of surgery and reduces the risk of corneal dehydration compared to conventional aaPRK method.^22^

## Conclusion

In conclusion, both tPRK and aaPRK are predictive and effective for the treatment of myopia and myopic astigmatism. Since both procedures provide similar postoperative CDVA, SE, asphericity, and HOAs in patients with myopia and compound myopic astigmatism, these two methods have no superiority over each other in terms of long-term results. When evaluated in terms of the aberration coefficient value, which is affected by all HOAs, aaPRK provides better results.

## Figures and Tables

**Table 1:**
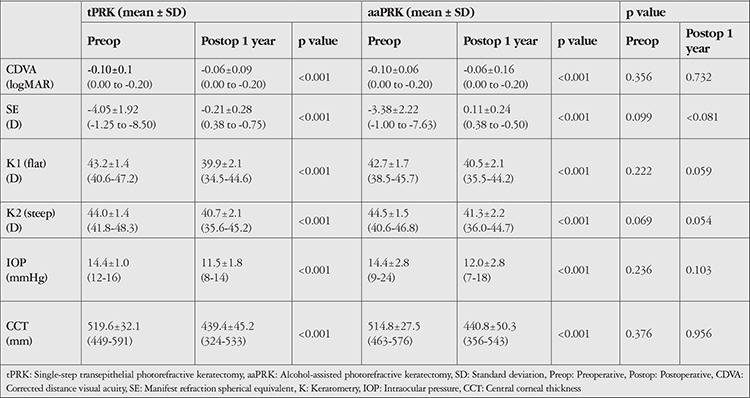
Comparison of preoperative and postoperative 1-year values of the tPRK (n=54) and aaPRK (n=54) groups

**Table 2:**
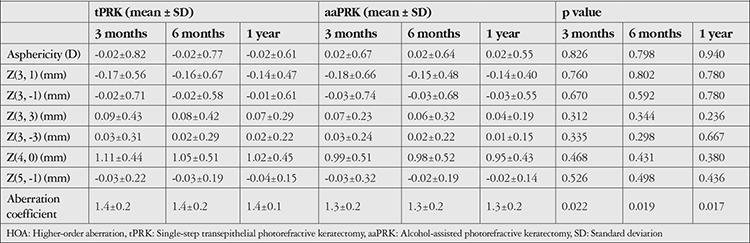
Postoperative asphericity and HOAs of the tPRK (n=54) and aaPRK (n=54) groups

## References

[1] Clinch TE, Moshirfar M, Weis JR, Ahn CS, Hutchinson CB, Jeffrey JH (1999). Comparison of mechanical and transepithelial debridement during photorefractive keratectomy. Ophthalmology..

[2] Carr JD, Patel R, Hersh PS (1995). Management of late corneal haze following photorefractive keratectomy. J Refract Surg..

[3] Lee HK, Lee KS, Kim JK, Kim HC, Seo KR, Kim EK (2005). Epithelial healing and clinical outcomes in excimer laser photorefractive surgery following three epithelial removal techniques: mechanical, alcohol, and excimer laser. Am J Ophthalmol..

[4] Aslanides IM, Padroni S, Arba Mosquera S, Ioannides A, Mukherjee A (2012). Comparison of single-step reverse transepithelial all-surface laser ablation (ASLA) to alcohol-assisted photorefractive keratectomy. Clin Ophthalmol..

[5] Luger MH, Ewering T, Arba-Mosquera S (2012). Consecutive myopia correction with transepithelial versus alcohol-assisted photorefractive keratectomy in contralateral eyes: one-year results. J Cataract Refract Surg..

[6] Fadlallah A, Fahed D, Khalil K, Dunia I, Menassa J, El Rami H, Chlela E, Fahed S (2011). Transepithelial photorefractive keratectomy: clinical results. J Cataract Refract Surg..

[7] Stojanovic A, Chen S, Chen X, Stojanovic F, Zhang J, Zhang T, Utheim TP (2013). One-step transepithelial topography-guided ablation in the treatment of myopic astigmatism. PLoS One..

[8] Murta J, Martins A (2012). Measurement and topography guided treatment of irregular astigmatism. In: Goggin M, ed. Astigmatism-Optics, Physiology and Management. Rijeka, Croatia: InTech Open Access Publisher.

[9] Kaiserman I, Sadi N, Mimouni M, Sela T, Munzer G, Levartovsky S (2017). Corneal breakthrough haze after photorefractive keratectomy with mitomycin C. Cornea..

[10] Mrochen M, Kaemmerer M, Mierdel P, Seiler T (2001). Increased higher-order optical aberrations after laser refractive surgery: a problem of subclinical decentration. J Cataract Refract Surg..

[11] Moreno-Barriuso E, Lloves JM, Marcos S, Navarro R, Llorente L, Barbero S (2001). Ocular aberrations before and after myopic corneal refractive surgery: LASIK-induced changes measured with laser ray tracing. Invest Ophthalmol Vis Sci..

[12] Seiler T, Kaemmerer M, Mierdel P, Krinke HE (2000). Ocular optical aberrations after photorefractive keratectomy for myopia and myopic astigmatism. Arch Ophthalmol..

[13] Sin S, Simpson TL (2006). The repeatability of corneal and corneal epithelial thickness measurements using optical coherence tomography. Optom Vis Sci..

[14] Kim WJ, Shah S, Wilson SE (1998). Differences in keratocyte apoptosis following transepithelial and laser-scrape photorefractive keratectomy in rabbits. J Refract Surg..

[15] Ozdamar A, Aras C, Okar I, Bahcecioglu H, Ozkan S (2000). Scanning electron microscopy comparison of mechanical and laser corneal epithelial removal techniques during photorefractive keratectomy. Turk J Ophthalmol..

[16] Ghoreishi M, Attarzadeh H, Tavakoli M, Moini HA, Zandi A, Masjedi A, Rismanchian A (2010). Alcohol-assisted versus mechanical epithelium removal in photorefractive keratectomy. J Ophthalmic Vis Res..

[17] Kaluzny BJ, Cieslinska I, Mosquera SA, Verma S (2016). Single-Step Transepithelial PRK vs Alcohol-Assisted PRK in Myopia and Compound Myopic Astigmatism Correction. Medicine (Baltimore)..

[18] Fattah MA, Antonios R, Arba Mosquera S, Abiad B, Awwad ST (2018). Epithelial Erosions and Refractive Results After Single-Step Transepithelial Photorefractive Keratectomy and Alcohol-Assisted Photorefractive Keratectomy in Myopic Eyes: A Comparative Evaluation Over 12 Months. Cornea..

[19] Antonios R, Abdul Fattah M, Arba Mosquera S, Abiad BH, Sleiman K, Awwad ST (2017). Single-step transepithelial versus alcohol-assisted photorefractive keratectomy in the treatment of high myopia: a comparative evaluation over 12 months. Br J Ophthalmol..

[20] Muller-Pedersen T, Cavanagh HD, Petroll WM, Jester JV (1998). Corneal haze development after PRK is regulated by volume of stromal tissue removal. Cornea..

[21] Helena MC, Baerveldt F, Kim WJ, Wilson SE (1998). Keratocyte apoptosis after corneal surgery. Invest Ophthalmol Vis Sci.

[22] Celik U, Bozkurt E, Celik B, Demirok A, Yilmaz OF (2014). Pain, wound healing and refractive comparison of mechanical and transepithelial debridement in photorefractive keratectomy for myopia: results of 1-year follow-up. Cont Lens and Anterior Eye..

